# Maternal-Fetal Perinatal Transmission of Staphylococcal Infections: A Report of Two Neonates

**DOI:** 10.1155/2020/8886049

**Published:** 2020-06-16

**Authors:** V. Thadchanamoorthy, Kavinda Dayasiri

**Affiliations:** ^1^Department of Pediatrics, Faculty of Health Care Science, Eastern University, Chenkalady, Sri Lanka; ^2^Department of Pediatrics, Base Hospital, Maha Oya, Sri Lanka

## Abstract

Staphylococcal infection in terms of fetus is rare and is associated with either maternal staphylococcal sepsis or colonization that leads to vertical transmission. Antenatal invasive procedures are a recognized risk factor. Most reported newborns with fetal-onset staphylococcal infections have had a worse outcome. We report the story of two newborns who had pustular-bullous skin lesions at birth and responded successfully to antistaphylococcal antibiotics. Both neonates made complete recovery. It is important to suspect clinically the staphylococcal infections when bullous skin lesions are present in the newborn.

## 1. Introduction

*Staphylococcus aureus* is a Gram-positive bacterial pathogen which is found on skin and has a vaginal colonization rate of 15% [1]. However, it is well known to cause severe soft tissue infections, and there is growing evidence that it can also cause chorioamnionitis leading to neonatal morbidity and preterm birth [2]. Maternal chorioamnionitis and staphylococcal neonatal septicemia are associated with worse outcomes and high neonatal mortality [3]. Authors report two newborns who had pustular-bullous skin lesions at birth without evidence of clinical chorioamnionitis and responded successfully to antistaphylococcal antibiotics. Both neonates made complete recovery. Prompt treatment with appropriate antistaphylococcal antibiotics leads to recovery without progressing to bacteremia and its complications like septic shock, meningitis, and septic arthritis.

## 2. Case 1

**Figure 1:**
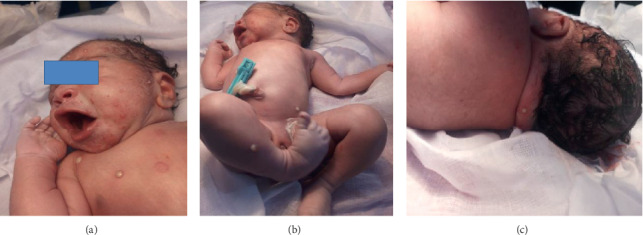
Extensive generalized pustular-bullous lesions at birth.

A newborn baby was admitted following detection of generalized pustular-bullous lesions at birth (Figure 1). She was born following emergency LSCS (lower segment cesarean section) due to lack of progress. She was the fourth child of a mother whose pregnancy was otherwise uncomplicated. Perinatal period was uncomplicated, and membranes ruptured only three hours prior to delivery following spontaneous labour. The mother also did not have fever or smelly liquor. Delivery was not assisted by instrumentation, and the baby was otherwise healthy with satisfactory APGAR scores.

She had septic screen at birth following which intravenous (IV) cloxacillin 50 mg/kg twice daily was commenced as for staphylococcal sepsis. Investigations revealed FBC-WBC 35 × 10^3^, neutrophils 84%, lymphocytes 12%, monocytes 5%, and CRP 12 mg·dl. CSF studies were normal. Both surface swab and blood cultures revealed *Staphylococcus aureus*. However, her skin lesions became extensive while on IV cloxacillin with poor response. Therefore, antibiotics were changed to IV meropenum 30 mg/kg every 8 hours. However, on meropenum, she made a gradual recovery with the resolution of bullous lesions, and meropenum was continued for 10 days. *Staphylococcus aureus* was sensitive to meropenum, cotrimoxaole, and linezolid. Although the mother was screened for MSSA (methicillin-sensitive *Staphylococcus aureus*) and MRSA (methicillin-resistant *Staphylococcus aureus*), her investigation results were normal.

## 3. Case 2

**Figure 2:**
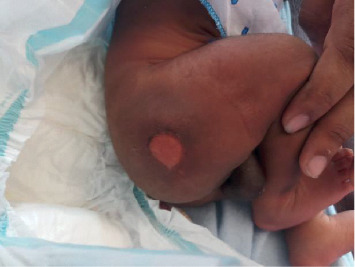
Bullous lesion over the buttock following rupture.

A term newborn baby who was delivered vaginally as a home delivery by a birth attendant was transferred to the local hospital for further management of a bullous lesion in the buttock noticed at birth (Figure 2). The mother was single and did not follow-up at an antenatal clinic. She did not have prolonged rupture of the membrane, pyrexia, or smelly liquor. Following admission, the baby developed poor sucking and became less active. She was commenced on intravenous cloxacillin 50 mg/kg twice daily following septic screening. Investigations revealed normal white cell count, and CRP was elevated (24 mg/dL). CSF studies were normal. Surface swab culture of the bullous lesion revealed *Staphylococcus aureus*. Blood culture however revealed no growth. IV cloxacillin was continued for seven days, and she had a gradual recovery while on antibiotics. Although the mother was screened for MSSA (methicillin-sensitive *Staphylococcus aureus*) and MRSA (methicillin-resistant *Staphylococcus aureus*), her investigation results were normal.

## 4. Discussion

Maternal staphylococcal colonization is associated with increased risk of staphylococcal infection in the newborn [4]. This report is unique that both reported newborns had established staphylococcal skin infections prior to birth indicating maternal-fetal transmission of the infection likely secondary to maternal vaginal colonization. Studies have shown that maternal colonization is associated with fivefold higher risk of neonatal colonization within 2 hours of birth [4]. However, direct mother to neonate transmission is rare [5]. Furthermore, the fact that the newborn reported under case 1 was delivered by LSCS suggests that she had staphylococcal bullous lesions in utero. Delivery by LSCS is associated with significant reduction in colonization of the newborn [6].

A previously reported case series of maternal-fetal staphylococcal infections identified their risk factors and complications in the newborn [7]. Among 11 children reported, the majority was preterm, and antenatal invasive procedures were a risk factor. Blood cultures were positive only in nine children. In their series, all children had a worse clinical course with respiratory and haemodynamic failure; however, the sample was restricted to newborns who were in the intensive care unit.

The pathophysiology of staphylococcal skin and soft tissue infections is related to secretion of bacterial extracellular proteins and bacterial surface components [8]. Exfoliative toxins A and B induce massive increase of T lymphocytes that secrete lymphokines IL-1, IL-6, and TNF-alpha [9]. Exfoliative toxin A further leads to formation of blisters. Blisters that are left untreated lead to bacteremia [10], and full recovery usually takes 2-3 weeks following commencement of appropriate antibiotics.

The differential diagnosis of vesiculobullous lesions in the newborn is numerous [11]. Infective aetiologies include bullous impetigo and herpes simplex infections. Inherited causes include hereditary epidermolysis bullosa and ichthyosis bullosa, while immunobullous disorders include neonatal bullous pemphigoid and linear IgA dermatosis. Careful clinical evaluation and microbiological demonstration of the pathogen are helpful in distinguishing neonatal staphylococcal bullous lesions from other etiologies.

Presenting clinical features of neonatal staphylococcal infections depends on site and severity of infection. Complications of neonatal staphylococcal infections include septic shock, pneumonia and lung abscesses, septic ileus and necrotizing enterocolitis [12], cerebral abscesses and meningitis, osteomyelitis, orbital cellulitis, septic arthritis, and endocarditis and are associated with high mortality [13].

Management of invasive staphylococcal infections includes intravenous antibiotics and treatment of associated complications. Uncomplicated *Staphylococcus aureus* bacteria need at least 10–14 days of intravenous antibiotics, whilst prolonged course of antibiotics is indicated for complications such as septic arthritis, osteomyelitis, meningitis, and brain abscesses.

## 5. Conclusion

It is important to clinically suspect staphylococcal infections when bullous skin lesions are present in the newborn. Blood cultures, however, may not reveal the pathogen in all cases, especially in the well newborn. Prompt treatment with appropriate antistaphylococcal antibiotics leads to the resolution of clinical features and recovery.

## Data Availability

Data associated with this report are available from the corresponding author on reasonable request.
